# Trajectories of multimorbidity: exploring patterns of multimorbidity in patients with more than ten chronic health problems in life course

**DOI:** 10.1186/s12875-014-0213-6

**Published:** 2015-01-22

**Authors:** Rein Vos, Marjan van den Akker, Jos Boesten, Caroline Robertson, Job Metsemakers

**Affiliations:** School for Public Health and Primary Care (CAPHRI), Maastricht University, Maastricht, The Netherlands; Department of Family Medicine, School for Public Health and Primary Care (CAPHRI), Maastricht University, Maastricht, The Netherlands; Department of General Practice, Catholic University Leuven, Leuven, Belgium

**Keywords:** Multimorbidity, Life time prevalence, Chronic health problems, Illness trajectories, General practice, Intensive forms of multimorbidity, Susceptibility

## Abstract

**Background:**

Physicians are frequently confronted with complex health situations of patients, but knowledge of intensive forms of multimorbidity and their development during life is lacking.

This study explores patterns and trajectories of chronic health problems of patients with multimorbidity particularly those with more than ten conditions and type and variety of organ systems involved in these patterns during life.

**Method:**

Life time prevalence patterns of chronic health problems were determined in patients with illness trajectories accumulating more than ten chronic health problems during life as registered by general practitioners in the South of the Netherlands in the Registration Network Family Practices (RNH).

**Results:**

Overall 4,560 subjects (5%) were registered with more than ten chronic health problems during their life (MM11+), accounting for 61,653 (20%) of the 302,808 registered health problems in the population (N = 87,837 subjects). More than 30% accumulates 4 or more chronic health conditions (MM4-5: 4–5 conditions (N = 14,199; 16.2%); MM6-10: 6–10 conditions (N = 14,365; 16.4%).

Gastro-intestinal, cardiovascular, locomotor, respiratory and metabolic conditions occur more frequently in the MM11+ patients than in the other patients, while the nature and variety of body systems involved in lifetime accumulation of chronic health problem clusters is both generic and specific. Regarding chronic conditions afflicting multiple sites throughout the body, the number of neoplasms seems low (N = 3,592; 5.8%), but 2,461 (49%) of the 4,560 subjects have registered at least one neoplasm condition during life. A similar pattern is noted for inflammation (N = 3,537, 78%), infection (N = 2,451, 54%) and injury (N = 3,401, 75%).

**Conclusion:**

There are many challenges facing multimorbidity research, including the implementation of a longitudinal, life-time approach from a family practice perspective. The present study, although exploratory by nature, shows that both general and specific mechanisms characterize the development of multimorbidity trajectories. A small proportion of patients has a high number of chronic health problems (MM11+) and keeps adding health problems during life. However, GP’s need to realise that more than one third of their patients accumulate four or more chronic health problems (MM4-5 and MM6-10) during life.

## Background

Multimorbidity (MM) is the co-occurrence of two or more diseases in a single individual [1,2]. Multimorbidity is a highly prevalent health problem and may be a heavy burden on the patient leading to adverse health effects, non-adherence to care, improper use of medication, and prolonged recovery time [3-8]. General practitioners are increasingly confronted with multimorbidity, resulting in complex care, where one condition might cause, maintain or exacerbate other conditions, affecting quality of life and leading to increasing use of health services [9,10].

Although primary care physicians treat patients with multiple health conditions on a daily basis, valid figures about prevalence of multimorbidity are scarce. Most studies report an increasing prevalence with age, but the figures vary widely due to different patient populations, study settings, definitions of multimorbidity (i.e., the medical conditions taken into account and the number of medical conditions) [11,12]. Health care systems may affect prevalence rate through organisational factors or the use of active protocols for detecting certain diseases. However, most estimates from primary care, which relied on practitioners records as data source, reported prevalence rates for multimorbidity between 20-30% for the entire population, and 50-90% for the elderly [11]. For inpatients those estimates are even higher [13].

While a large literature concerns prevalence, the prediction of risk and prognosis of co-occurring diseases in multimorbidity patients, relatively little research is concerned with the course of illness during life in patients with multimorbidity. Most multimorbidity studies focus on the identification of specific combinations in patient populations, based on one index disease and additional conditions, either in general or specific population-based studies or in administrative databases [14-17]. Different measures of multimorbidity have been used, ranging from simple counts of the number of diseases or clusters of diseases, and the number of medications, up to severity measures like the Charlton index which differentially weight diseases. Such measures are useful in both epidemiological and experimental studies of interventions in primary care or in measuring the outcome [18].

In recent years a few studies have appeared to investigate multimorbidity patterns, using data mining techniques, e.g. factor analysis methods, to investigate clusters of health problems. These studies are cross-sectional and investigate prevalence patterns in specific age groups, and restrict analyses to a limited number of health conditions [19-22]. A life span perspective is missing and there is a lack of understanding of trajectories of multimorbidity in the course of life of patients in primary care.

The concept of distinct trajectories of illness over time is well established in other advanced, medical specialty areas [23-25]. Longitudinal analysis of multimorbidity, however, is complex. Varying definitions of multimorbidity exist and different scopes of time windows are abound [26,27]. In this study we transcend the definition of multimorbidity of two or more specific conditions and move from the single disease paradigm to a broad, system-theoretic approach of multimorbidity [28-31]. Taking into account the body of knowledge, it is still not understood why many patients get one health problem after another, whereas others are hardly ever afflicted. So far, there is no satisfying explanation for obvious differences in disease susceptibility [32,33]. However, research into biological, behavioral, psychological, and demographic factors and their relation to health status has led to the theory of general disease susceptibility [34,35]. This theory states that sociological, psychological, genetic, and immunological factors are underlying factors that influence susceptibility to a wide range of conditions. They are general rather than specific risk factors. This indicates that it might be worthwhile to incorporate the wide range of possible conditions and their interactions in the study of trajectories of multimorbidity [36].

Previous studies focus on specific chronic conditions and restrict comorbidities to lists of more common chronic conditions [2,9,30]. In this way the focus is on specific risk factors only, not on general factors. These general conditions, mostly excluded from the studied comorbidities, are important for three reasons. First, they have implications for health management, patient education and outcomes. Second, these additional, general health problems are also important in the development of multimorbidity as shown in a previous cohort-study, − listed as one of the mere six cohort studies listed in a recent world-wide review of 996 screened articles on multimorbidity [9]. Third, general disease susceptibility and its relationship to the development of multimorbidity is associated with psychosocial characteristics, such as an internal locus of control, an active coping style, a palliative coping style, and the occurrence of positive life events. Limiting multimorbidity to specific lists of health problems might underestimate the influence of psychological and social factors on patient’s illness trajectories [36].

This study therefore aims to describe patterns and trajectories of chronic health problems of patients with multimorbidity, particularly those with 11+ conditions, and the types and variety of organ systems involved during the life course. This broad view on chronic health conditions fits the perspective and position of the family physician as a health professional with a complete overview of the patient and his or her family and social context. Multimorbidity will be investigated by exploring underlying, but possibly connecting structures and processes between organ systems, e.g. inflammation, infections and injuries affecting multiple sites in the body.

We report an exploratory study of a sample of 4,560 (5%) subjects from the RNH-database (N = 87,837) with a large number of diseases, which comprise one extreme part of the Gaussian-distribution of multimorbidity, namely the accumulation of more than ten chronic health problems in their lifetime, as registered by general practitioners.

## Methods

### Context

This study was carried out in the context of the Registration Network Family Practices (RegistratieNet Huisartspraktijken, RNH). The RNH is a continuous database, in which about 70 general practitioners (GPs) working in 22 different practices in the South of the Netherlands are participating. All relevant health problems are registered. A health problem is defined as ‘anything that has required, does or may require health care management and has affected or could significantly affect a person’s physical or emotional well-being’ [37,38]. The GPs systematically collect and register all health problems, which are coded in a standardized fashion, according to the International Classification of Primary Care (ICPC), following the criteria of the International Classification of Health Problems in Primary Care (ICHPPC-2) [39]. ICPC is a diagnostic classification developed under the umbrella of WONCA and the WHO, with relations to other diagnostic classifications such as the ICD [39,40]. In complex medical conditions registration is almost always based on a specialist diagnosis reported to the GP. In the Netherlands, GPs have comprehensive information on the health status of their patients because GPs function as gatekeepers to other health care facilities and it is compulsory for all Dutch residents to have health care insurance and to register with a GP. The GPs add the registered health problems to the general RNH database, not the source of the diagnostic information. However, only health problems fulfilling the ICHPPC criteria are listed by the GP in the general database. Every three months the coded health problems are transferred to the RNH database by the GP. When patients are newly enrolled in a practice, significant morbidity is retrospectively entered in the electronic medical record, thus enabling to have a comprehensive view of patients health status throughout their lives [41].

A special note deserves the conversion of the handwritten records of the GPs before 1990, when the general computerized database started [42,43]. In all participating practices computerized health information systems were installed, replacing the handwritten records. Data on patient encounters and other health information are stored on the computer. On a daily basis, the general practitioners complete the records of several patients, by adding patient characteristics and revising the problem list. It was stipulated that the general practitioners should not develop a specific pattern such as first completing the records of elderly patients or patients with asthma, but should ‘randomly’ select patients. Once completed, the data for a patient is kept up to date. Aspects of problem definition and coding were discussed in ‘consensus groups’. Five such groups, of about eight general practitioners each, have been formed, meeting four or five times a year. In the past ten years the results of the consensus groups have been integrated in automatic software tools. The GPs of the participating family practices are still involved in the academic network. Despite the extensive process of control and auditing it is clear that the conversed codes of the handwritten records were accepted without checking ICHPPC criteria, since details of the medical history were no longer available in many cases [43].

Membership of the RNH population ends by migration or death. All patients included in the RNH database have been informed about the anonymous use of their health information and their medical information is not incorporated in the database if desired. The quality of the data is ascertained by ample instruction and training sessions, regular regional consensus groups, quality control audits, an online thesaurus available during data-entry and systematic control [43,44]. Participating GPs are used to and trained in structured and automated registration of health problems using ICPC codes, and have additional computer facilities for registration and coding. The RNH population is comparable to the Dutch population with regard to socio-demographic variables. This renders the RNH a valid and precise database for medical research. Since 1990 there were more than 60 research projects with an yearly average of two PhD-theses and 8 international publications making use of the data and the infrastructure of RNH [44].

#### Included health problems

To avoid meta-discussions on the concepts of health and disease, for example, whether depression or pregnancy-related conditions should be considered as ‘disease’ , the present study addresses the notion of health problems as used in RNH following the ICPC classification as described above. For this study only the ICPC codes related to diagnostic categories were selected. ICPC codes representing symptoms and complaints were not selected. Furthermore, for calculating the occurrence of multimorbidity a number of ICPC codes were not selected, because they represent pregnancy and delivery without pathology, test results not leading to a diagnosis, variation of normal function and risk factors. In our database only the subset of *chronic* health problems [37,38], which have been recorded by the GP, when they have lasting consequences for the functional status or prognosis of the patient were selected. Only permanent problems (no recovery expected), chronic problems (duration longer than six months), recurrent problems (more than three recurrences within a period of six months) and problems with a high impact on daily functioning are included. Notably, problems are stated at the highest level of refinement that can be reasonably defended. That is, a condition cannot be given two problem definitions at the same time. However, different health problems can be assigned within a certain time window. This implies registration at different dates by the GP related to subsequent visits by the patient to the GP. In this study the first year of registration of the chronic health problems by the GP was included. Sixteen of the seventeen ICPC Chapters containing diagnostic codes related to chronic health problems were included: A (General), B (Blood, D (Digestive), F (Eye), H (Ear), K (Circulatory), L (Locomotor), N (Neurological), P (Psychiatric, psychological), R (Respiratory), S (Skin), T (Endocrine, metabolic and nutritional), U (Urinary conditions), W (Pregnancy related health problems), X (Female conditions), Y (Male conditions). ICPC codes representing symptoms and complaints were not selected. A total of 335 different diagnostic codes was included for the analysis. Data stem from the Registration Network Family Practices database, available on July 1^st^, 2010 and consisted of 87,837 individuals of all ages.

#### Definitions

Multimorbidity is the occurrence of two or more health problems within one person [41]. The time window chosen is both cross-sectional and longitudinal: the selected MM11+ subjects have at least an episode of two or more health problems diagnosed within one year by the GP; and they have a course of life trajectory of multiple, namely 11 or more health problems. For every subject, each health problem was assessed for both lifetime and past-12-month history. Lifetime prevalence refers to the proportion of the population that has ever fulfilled diagnostic criteria, i.e., experienced a certain number of health conditions at some time in their life. Life time prevalence of health problems was calculated as the number of subjects who had none, one, two, three, and up to eleven-and more conditions divided by the total number of persons in the RNH-database (N = 87,837). The emphasis of this study is the group of patients with more than ten chronic health problems in life course. However, the dynamics is of interest as well: patients develop a number of health problems during their life, from few health problems in some patients to more or many health problems in other patients. For this reason we also made a comparison of subgroups of patients with four to five (MM4-5), six to ten (MM6-10), and more than ten (MM11+) conditions. The basic subject characteristics used in the analysis were: sex (males and females), age in five categories (0–19, 20–39, 40–59, 60–79, 80 years and older), and year/time span of new diagnoses of the GP.

#### Analysis

Descriptive statistical methods were used to determine the number of health problems per subject for the total RNH-population as well as for the multimorbidity subgroups of four to five (MM4-5), six to ten (MM6-10), and more than ten (MM11+) health problems. This analysis was related to the basic patient characteristics used: sex (males and females) and age in five categories (0–19, 20–39, 40–59, 60–79, 80 years and older). Lifetime prevalence was analysed for the separate health problems – the 335 ICPC codes – as well as for the sixteen ICPC-Chapters. The ICPC chapters can be considered as a classification system of health conditions in terms of their location in and impact on organ systems. Following our system-theoretic approach, we added a third aspect. Health conditions may also have impact on different physiological processes throughout the body. For example, inflammation, infections and injuries may affect multiple sites in the body. The ICPC codes have been categorized in seven classes: *inflammation* (I) denoting some kind of inflammation process internally in the body, *infection* (II) denoting an episode of chronically being inflicted by an infectious agent external to the body, *injury* (III) denoting an event of damage by intrusion of an object or procedure external to the body, including complications or adverse effects of medical actions, *specific-conditions* (IV) denoting ‘classic’ diagnostic categories in the respective ICPC chapters ranging from, for example, asthma and COPD in the respiratory ICPC chapter to angina pectoris and myocardial infarction in the cardiovascular ICPC chapter –, *neoplasm* (V) denoting all forms of neoplasm, including benign tumours, but we also used a sub-classification scheme separating the malignant carcinomas and the nonspecific or benign tumours throughout the ICPC chapters, *congenital* (VI) denoting diseases or dysfunctions at birth, and *otherwise* (VII) denoting either the 99-categories of the ICPC chapters or miscellaneous ICPC codes not covered by the previous six categories. The classification was performed by the GPs of the team (JB, CR, JM); in cases of doubt the category “otherwise” was used, e.g. when the border-line between inflammation and infection is unclear such as B70 “acute lymphadenitis”, which might be an inflammation process without, but also due to a present infection; or when mixed ICPC-codes were at stake, e.g. codes that combine different conditions, e.g. D83 and D84 (“Disease of mouth/tongue/lips” respectively “Disease of oesophagus”). Lifetime prevalence for these categories of conditions was also calculated as described above.

#### Ethical approval

The study was conducted in compliance with Good Clinical Practice guidelines Procedures, the principles of the Declaration of Helsinki (version October 2008) and the Dutch (Medical Research Involving Human Subjects Act and Personal Data Protection Act) law. The privacy regulation of the study was registered at the Dutch Data Protection Authority. According to Dutch legislation, neither obtaining informed consent nor approval by a medical ethics committee was obligatory for observational studies.

## Results

### General aspects: demographic factors

The RNH population accounts for 87,837 subjects with a total of 302,808 registered chronic health problems. Overall 4,560 subjects were found as being registered with more than ten health problems in their life time (MM11+), accounting in total for 61,653 conditions, which is on average 13.5 health problems per subject. The MM11+ group consists of 5% of the subjects accounting for 20% of the registered health problems (Table 1).

**Table 1:** **Descriptive characteristics of the RNH-population**

**CHP per subject**	**Number of subjects (%)**		**Number of conditions**	**CHP/subj average**	**Column-%**
**0 = MM0**	17,273	(19.7)	0	0	0
**1 = MM1**	14,328	(16.3)	14,328	1	4.7
**2 = MM2**	12,762	(14.5)	25,524	2	8.4
**3 = MM3**	10,350	(11.8)	31,050	3	10.3
**4-5 = MM4-5**	14,199	(16.2)	62,939	4.4	20.8
**6-10 = MM6-10**	14,365	(16.4)	107,314	7.5	35.4
**11+ = MM11+**	4,560	(5.2)	61,653	13.5	20.4
**Total**	87,837	(100,1)	302,808	3.4	100

CHP = chronic health problems; MM = multimorbidity, the digits denoting the number of chronic health problems.

Regarding basic patient characteristics the number of chronic health problems is particularly related to age. Whereas there are merely 71 cases of MM11+ in the age groups 0–19 (N = 7) and 20–39 years (N = 64), the number of cases of MM11+ rapidly increases with age, with a ‘peak’ of 2,518 subjects (55%) in the age group of 60–79 years (Table 2). It can be noted that the peak of the MM4-5 patients is in the age-group of 40 to 59 years (N = 5,710, 40%). The MM6-10 group shows a trend intermediate between the MM4-5 and MM11+ subgroups.

**Table 2:** **Age characteristics of RNH population**

**Age**	**MM4-5**	**%-column**	**MM6-10**	**%-column**	**MM11+**	**%-column**	**RNH-All**	**%-column**
**-0-19**	779	5	187	1	7	0	16,137	18
**-20-39**	2,750	19	1,215	9	64	1	18,937	22
**-40-59**	5,710	40	4,727	33	699	15	28,342	32
**-60-79**	4,355	31	6,593	46	2,518	55	20,250	23
**-80+**	605	4	1,643	12	1,272	28	4,171	5
**Total**	14,199		14,365		4,560		87,837	

[MM = multimorbidity, the digits denoting the number of chronic health problems].

There are important differences between men and women in the MM11+ group. More men are represented in the age group of 60–79 years than in the age group of 80 years and older; this trend is mitigated for women: they also have a peak in the age of 60–79 years, but the decline of the age group of women aged 80 years and older is relatively less dramatic than for men because women are represented two times more than men in the age group of 80 year and older (see Table 3).

**Table 3:** **Sex characteristics of RNH population (N = 87,837)**

	**MM11+**				**RNH-All**			
	**Male**		**Female**		**Male**		**Female**	
**Age**	**Number**	**%-row**	**Number**	**%-row**	**Number**	**%-row**	**Number**	**%-row**
**0-19**	3	43	4	57	8,185	51	7,952	49
**20-39**	29	45	35	55	9,391	50	9,546	50
**40-59**	285	41	414	49	13,924	49	14,418	51
**60-79**	1,091	43	1,427	57	9,887	49	10,363	51
**80+**	405	32	867	68	1,454	35	2,717	65
**Total**	1,813	40	2,747	60	42,841	49	44,996	51

[MM = multimorbidity, the digits denoting the number of chronic health problems].

Although the number of 44,996 (51.2%) women in the total RNH-population is somewhat higher than for men (N = 42,841, 48.8%) – as is the case in the Dutch population in general -, the different age groups for men and women follow a similar trend in the total RNH-population.

### Range and scope of clustering of health problems and afflicted body systems

Two important patterns of multimorbidity can be identified. The first is that certain clusters occur more or less frequent in the MM11+ than in the other patients. The second pattern is the nature and variety of body systems involved in lifetime accumulation of health problem clusters.

With regard to the first pattern it can be noted that some health problem clusters as represented in the ICPC chapters are prominent in the MM11+ group – and in the other subgroups of multimorbidity -, in particular the locomotor, cardiovascular, gastro-intestinal, respiratory and metabolic health problems (see Table 4).

**Table 4:** **Distribution of chronic health problems (N = 302,808) in the ICPC chapters for different subgroups of multimorbidity**

**CHP-Group**	**MM4-5**	**Proportion**	**MM6-10**	**Proportion**	**MM11+**	**Proportion**
**General**	5,098	0,08	6,887	0,06	3,099	0,05
**Blood**	486	0,01	934	0,01	642	0,01
**Gastro-Int**	5,599	0,09	10,968	0,10	6,999	0,11
**Eye**	1,817	0,03	4,326	0,04	3,272	0,05
**Ear**	1,913	0,03	2,402	0,02	1,215	0,02
**Cardiovasc**	6,947	0,11	16,015	0,15	11,523	0,19
**Locomotor**	11,619	0,18	20,859	0,19	12,075	0,20
**Neurology**	2,718	0,04	4,484	0,04	2,557	0,04
**Psychiatric**	3,308	0,05	4,918	0,05	2,061	0,03
**Respiratory**	7,887	0,13	9,425	0,09	4,137	0,07
**Skin**	4,966	0,08	6,724	0,06	3,082	0,05
**Metabolic**	4,570	0,07	9,208	0,09	5,449	0,09
**Urinary**	1,299	0,02	2,697	0,03	1,855	0,03
**Pregnancy**	794	0,01	950	0,01	300	0,00
**Female**	2,589	0,04	4,487	0,04	2,412	0,04
**Male**	1,329	0,02	2,030	0,02	975	0,02
**Total**	62,939		107,314		61,653	

[CHP = chronic health problem; MM = multimorbidity, where the digit denotes number of chronic health problems].

If we just look at the occurrence of health conditions within the organ systems as represented by the ICPC chapters, there are clear shifts in the prevalence for the different subgroups of multimorbidity in general practice. This is particularly the case for the cluster of cardiovascular conditions, which increases from 11% of all disease conditions in the MM4-5 group, through 15% in the MM6-10 to 19% in the MM11+ group. The cluster of locomotor, gastro-intestinal health problems and eye conditions show a similar, albeit more slowly, increasing pattern. Some clusters, however, exhibit a *reverse* pattern: such a decreasing trend can be noted for the respiratory conditions (ICPC Chapter R): from 13% in the MM4-5 and 9% in the MM6-10 group to 7% in the MM11+ group. A similar trend, but less dramatically, is shown for the patients with psychiatric conditions, skin health problems and general conditions.

**Figure 1:**
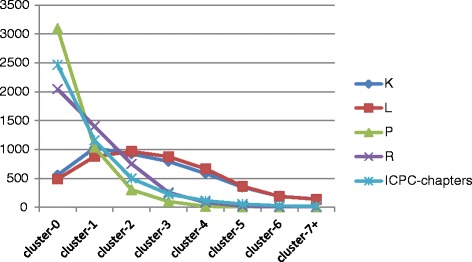
**Distribution of number of chronic health problems within the ICPC chapters counted for each patient (Lifetime) in MM11 + −group (N = 4,560).** For each patient in the MM11+ group the number of chronic health conditions during life are counted for each ICPC chapter separately. The term ‘cluster’ indicates the number of chronic health problems per subject WITHIN a particular ICPC chapter. For example, ‘cluster-3’ means that a patient has clustered three health conditions within one specific ICPC chapter in his or her trajectory, whereas ‘cluster-7+’ means that a patient has clustered 7 or more health conditions within one specific ICPC chapter. An individual patient may have clusters of different sizes, e.g. a certain patient may have a cluster of three respiratory conditions as well as a cluster of 7 or more gastrointestinal conditions. The capital letters K, L, P and R denote the ICPC chapters for cardiovascular, locomotor, psychiatric and respiratory conditions respectively, whereas the term “ICPC-chapters” refers to the calculated average for all 16 ICPC chapters; the y-axis shows the number of subjects in the MM11 + −group (N = 4,560) with the respective clusters of health conditions.

However, different dynamics can be distinguished when inspecting the trajectories of the individual patients. Some patients develop more diseases within one organ system, which underlies, the age-related changes in the pattern of multimorbidity. This is particularly the case for the ICPC chapters of cardiovascular (K) and locomotor (L) conditions (see Figure 1).

When afflicted by cardiovascular or locomotor conditions the number of health problems *within* these organ systems for an individual patient might increase during life, even up to seven or more conditions as in 142 patients with cardiovascular conditions and in 138 patients with locomotor conditions. In contrast, there are few patients with 7 or more chronic conditions in the other organ systems – on average for each organ system only 19 patients or less (Figure 1).

Figure 1 shows this differential dynamics: patients cluster more health conditionswithin the ICPC chapters of cardiovascular and locomotor conditions – with a broad ‘top’ at two, three and four conditions pro patient. In contrast, the number of conditions in the mental, respiratory and other organ systems – the ICPC chapters P, R and ‘other’ (Figure 1) – pro patient are rapidly declines from 1 to 2 or more. This indicates less clustering of health conditions in these organ systems for patients individually.

However, it is important to note that in total 3,393 (74.4%) of the 4,560 patients have 4 or more conditions within one organ system, although most patients have a ‘broad’ or ‘flat’ profile as well: these patients accumulate a broad range of conditions in their body systems.

### Patterns of multimorbidity involving infringements in-between organ systems

Several chronic conditions may originate in a particular organ system, but still may strain or afflict other organ systems or multiple sites throughout the body. Regarding the categorized processes, in particular inflammation, infection, injury, and neoplasms, some interesting patterns can be indicated (Table 5).

**Table 5:** **Distribution of Chronic Health Problems (CHP) related to categories of health problems In-Between ICPC chapters in the MM11+ group N = 4,560 subjects, N = 61,653 chronic health problems**

				**Number**		**Patients**	
**Category**	**Type**			**CHP**	**%**	**(N = 4,560)**	**%**
**I**	Inflammation			6,987	11.3	3,537	77.6
**II**	Infection			3,629	5.9	2,451	53.8
**III**	Injury			5,556	9.0	3,401	74.6
**IV**	Specific conditions			32,016	51.9	n.c.	
**V**	Neoplasms			3,592	5.8	2,461#	54
		Maligne	1,444				1,219 (27%)
		Other-benign	2,148				1,758 (39%)
**VI**	Congenital			490	0.8	n.c.	
**VII**	Otherwise			9,383	15.2	n.c.	
**Total**	ALL-types			61,653	100		

[#denotes including the overlap between patients with the two subtypes of neoplasm, namely N = 516 (of the 1,219 + 1,758 subjects with respective subtypes); n.c. denotes ‘not calculated’].

Most (N = 32,016; 51.9%) chronic conditions concern the specific health problems. The second and third most prevalent categories concern the ‘other’ health problems (N = 9,383; 15.2%) and the inflammation conditions (N = 6,987; 11.3%), with a varying contribution by the other categories (Table 5). However, the frequency of the chronic conditions in the categories only provides a part of the picture. Noteworthy is that the neoplasm or tumour related conditions seem to score quite low in frequency (N = 3,592, 5.8% of all registered chronic health conditions), but in fact 2,461 (49%) of the 4,560 subjects have registered at least one neoplasm condition in their life time prevalence profile. Differentiating the patients with a code of neoplasm in their lifetime profile in two groups - the malignant, organ-related neoplasms (N = 1,444 health problems, 1,219 patients) and the unspecified or benign neoplasms (N = 2,148; 1,758 patients) show a similar pattern (See Table 5). Similarly, 3,537 (77.6%) subjects have included in their lifetime MM-profile an inflammatory code. This score is lower, but still considerably prominent for the categories of infection (N = 2,451 subjects, 53.8%) and injury (N = 3,401, 75%).

## Discussion

### Summary of main findings

In this paper we have studied a selective group of patients with trajectories of high numbers of multiple pathology. This concerned patients who accumulated eleven or more chronic health problems in lifetime as registered by the RNH-general practitioner. This intensive form of multimorbidity was defined as comprising one extreme part of the Gaussian-distribution of multimorbidity. Overall 4,560 subjects were registered with more than ten chronic health problems during their life (MM11+). This group comprises 5% of the RNH-patient population, but accounts for 61,653 (20%) of the 302,808 registered chronic health problems in the RNH population (N = 87,837 subjects). Some health problem clusters as represented in the ICPC chapters are prominent in the MM11 + −patients, in particular the locomotor, cardiovascular, gastro-intestinal, respiratory and metabolic health problems. It is important to note that in total 3,393 (74.4%) of the 4,560 patients develop four or more conditions within one organ system in the course of life. However, most patients have a ‘broad’ or ‘flat’ profile as well: patients accumulate a broad range of conditions in their body systems. Most patients in the MM11+ group accumulate two or more times during their life two or more chronic conditions in one year, with a variety of chronic conditions distributed over the organ systems as classified in the ICPC-chapters (data not shown). About half of the patients have been confronted with a neoplasm diagnosis, be it of a benign or malignant type. Patients with intensive forms of multimorbidity have very often been inflicted during their life by chronic health conditions related to infection, inflammation, and injury.

The presented results show that intensive forms of multimorbidity involve a broad range of organ systems. This indicates that next to specific also more general mechanisms may be at work. The approach used in this study expresses this twofold approach to health problems. On the one hand a specific approach investigating the specific health problems and relating this to the type and number of organ systems involved as represented by the ICPC chapters. On the other hand, a broad, system theory and non-specific approach, which embraces the perspective of general disease susceptibility [28,29,35,36]. Thus, chronic health problems were taken into account, which may affect multiple sites in the body due to strains and pressures “external” from outside the subject, e.g. injuries and infections, and “internal” within the subject, e.g. inflammations and tumours, − although originating in a particular organ system or specific site in the body as referred to by the separate ICPC codes.

#### Strengths and limitations of the study

This study is the first, as far as known by the authors, investigating the topic of intensive forms of multimorbidity in general practice from a lifetime prevalence perspective. We were able to collect for all included patients the dates of the new diagnosis during their lifetime and to construct the life time trajectories of the accumulation of chronic conditions for each patient individually. The data presented here are the result of the exploratory analysis used as a heuristics for getting a better understanding of the dynamic patterns involved, hence are aggregated at a group level comprising the patients with what we call intensive forms of multimorbidity.

This study suffers from several limitations. Firstly, the study comprises all the limitations inherent in any retrospective design which uses electronic medical records as a data source. We tried to compensate for the weaknesses of this way of data collection and analysis as much as possible as described above. The study is based on electronic patient records comprising various data, but in this study we focused on the diagnosis of the chronic health conditions, so that our analysis of the diagnoses as registered by the GPs would be reliable. The quality of the data is assured by instruction and training sessions, regional consensus groups, quality control experiments, and special software programs, such as an automated thesaurus and automated checking for erroneous or missing entries. Reliability and completeness have been proved previously [42-44]. It is important to stress that in the Netherlands the GPs have comprehensive information on the health status of their patients because GPs are the gatekeeper to other health care facilities, and it is compulsory for all Dutch residents to have health care insurance and to register with a GP. Further, GPs will be informed on a routine basis by clinical specialists in the hospital of the diagnosis and other medical relevant data of their patients.

Therefore we expect this study not to suffer too much from the limitations possibly implicated by underdiagnosis or misclassification, for example misclassification of COPD being diagnosed as asthma or other comparable cases in the diagnosis of chronic health conditions.

However, diagnostic habits may differ between GPs in different countries and regions within countries, due to differences in the level of professional training, the degree of implementation or content of clinical practice guidelines, the use of active protocols for detecting certain diseases, organisational factors, etc. [11,45,46]. For example, Aarts et al. [45] showed that 3 of the 21 practices involved in the RNH-network diagnosed a relatively high percentage of diabetes patients with depression (ranging from 9.5 – 9.8%), while in 3 of the 21 practices this percentage is lower (ranging from 4.0 to 4.7%). Notably, the GPs were not instructed to systematically screen patients for possible depression or depressive symptoms: which could have led to a lower risk estimate. It seems plausible that GPs differ regarding their inclination to diagnose a depression. After investigating the most important characteristics of the practices, such as geographic place (defined by postal code) of a general practice; total number of diagnosed depressive disorders in the general practice, number and gender of patients, education of the patients, and number and gender of GPs in a practice, the authors were not able to identify any specific characteristics that could explain this effect [45]. This diagnostic variability may have important implications for general practice. In the RNH a large number of GPs is participating and previous studies showed that a minimum number of 25 to 30 GPs is sufficient to take into account the inter-GP variety in coding [44].

Despite such differences in prevalence, diagnostic habit and health care organisation, relevant similarities of multimorbidity patterns can be found in different European regions, as in the north-east of Spain and the south-east of the Netherlands [46]. In addition, the Dutch general practice holds an outstanding position regarding quality assurance and guideline implementation with respect to other European countries [46,47]. This also endorses the use of primary care electronic medical records for the epidemiologic characterization of multimorbidity. The use of electronic medical records would enable a longitudinal approach to the multimorbidity phenomenon. Understanding the way in which health conditions are associated with one another throughout the lives of individuals, as well as knowing how frequently these diseases appear, will bring about a better understanding of multimorbidity.

Furthermore, a broad spectrum of 335 chronic health problems were analysed. This included codes which enable GPs to register chronic health problems, which they cannot define in a strict way – e.g., the 99-codes -, as the ICPC-classification aims to provide the whole spectrum of health problems in family practice. This study does not intend to measure prevalence of chronic conditions or groups of chronic conditions nor to determine risk factors as such. Lifetime prevalence rates or lifetime risks were not estimated. Instead, the study focuses on the dynamics of life time patterns of multimorbidity in patients. In this study we selected a specific group of patient with a life time prevalence of more than health conditions. We did not take, for example, a cohort of patients who have been identified with similar characteristics of multimorbidity and then followed them forward. Even then, longitudinal analysis accounting for the different temporal aspects, e.g., age, birth and cohort aspects, is complex by itself. We considered it useful to start with a relatively small proportion of the population made up of people with high numbers of health conditions throughout their life, comparing trends and patterns with this selective group and with the other groups of multimorbidity patients (MM4-5, MM6-10). The progression among these subgroups requires a more fine-grained investigation of transitions in lifetime multimorbidity. Such a more dynamic in-depth analysis goes beyond the scope of the current study.

Other limitations have to be noted. The classification scheme of inflammation, infection, tumours and injury must be considered carefully and no biological connection between these biological processes and the occurrence of health problems is established in this study. However, it is worthwhile to see how the accumulation of specific health problems may relate to broader patterns of morbidity. It is generally acknowledged that – apart from socioeconomic and demographic factors – a broader range of host response mechanisms, ranging from genetic factors, biological stress mechanisms and psychosocial processes, e.g. coping styles, social network of the patient, operate at the level of the individual, but have also an important influence at the population level [31,33,34,36].

The analysed patterns are interesting from the perspective of daily care by GPs. The study shows that a certain number of patients accumulate larger series of health problems in life. Although sometimes health problems may be some event in some period of life of a subject, the health problems analysed here are all events only coded and registered by the GP when they are permanent (no recovery expected), chronic (duration longer than 6 months) or recurrent (more than three recurrences within 6 months), or when they have lasting consequences for the functional status or prognosis of the patient. Thus, the health problems concern conditions with great impact on patient’s lives and daily care by GPs. Although these conditions do bear a great impact, it has to be noted that the burden of multimorbidity may differ for patients and their GPs. A patient with malignant cancer, heart failure and renal disease may only have these three comorbid conditions but their severity is significant. On the other hand some other patient may have mild depression, diet controlled diabetes and a dry skin and equally may be labelled as having three comorbid conditions. The intensity of a patient’s multimorbidity may vary on the progressive state of the conditions present and the specific conditions diagnosed [1,18]. The main focus of this study was not to assess severity of conditions and multimorbidity, but to disentangle multimorbidity trajectories and patterns, among those patients with a large number of chronic health problems occurring during life (MM11+).

#### Comparison with existing literature

Research into multimorbidity is relatively new, mostly encompassing population studies, hospital studies and primary healthcare studies. In recent years multimorbidity has received fortunately increasing attention addressing the issues related prevalence, determinants, consequences and the patterns of multimorbidity, in general as well in different age-groups [1,2,9,26]. This study is unique, as far as known by the authors, for investigating the topic of severe forms of multimorbidity in general practice from a lifetime prevalence perspective. For reasons of investigating both disease-specific and more general patterns of multimorbidity, referring to the literature on general disease susceptibility and psychological and social determinants of multimorbidity [36], we focused on a broad scope of chronic disease conditions. Most multimorbidity studies select a much smaller set of chronic diseases or regroup diagnoses into specific chronic disease groups, as for example in the Expanded Diagnosis Clusters (EDC) [48,49]. In this study we started from the daily practice of the GP, aiming to include all the chronic conditions as registered in their care for complex patients. Definitions of multimorbidity should both inform and reflect clinical practice. This objective may be difficult to achieve when epidemiology oriented definitions are less inclusive and aim at a limited set of clear-cut criteria. For ‘diseases’ with varying latency or a chronic course, such as multimorbidity, developing a definition depends on decisions regarding which phase to monitor – asymptomatic, early phase, late phase – and on the circumscription of the spectrum of morbidity [50,27]. In our study we made the decision to start from the health problems as addressed by the GPs themselves. Only ICPC codes (ICPC70-99) that correspond to serious or chronic diseases were entered into the database.Higher-level regrouping of diagnoses into diagnosis clusters is foreseen for future research.

#### Implications for research and daily care

Primary care is characterized by a heterogeneous patient population, which comprises, about 120 patients with intensive forms of multimorbidity per standard practice (of 2350 patients) as is usual in the Netherlands [51]. These patients representing the 5% extreme of the Gaussian distribution cover about 20% of all chronic health conditions. It should be added that it is not a black-white, stationary picture. The subgroup of multimorbidity patients with six to ten chronic conditions, encompass patients who can be indicated to develop rapidly an increasing number of conditions during certain periods in their lives. Further, most of the patients in both multimorbidity groups – i.e. the MM6-10 and MM11+ − exhibit new diagnoses up to the time of the data-collection (July 2010) for this study. In fact, it can be shown (data not presented in results) that almost half of the MM-11+ (N = 2010; 44%) have a new diagnosis added in one year before the data extraction and many ((N = 3815; 83.6%) MM11+ have at least two new diagnoses in the past five-year period (2005–2009) with a mean of 3.35 new diagnoses (95% CI 3.2 - 3.4; median = 3.0). This is to note the intrinsic dynamic pattern of the life time prevalence pattern of the multimorbidity patients. Considering the fact that the diagnoses concern merely the registered chronic health conditions, leaving out the symptoms, medications and other health care activities, this underlines that complex care is an intensive task for the GP. Given the GPs’ expertise in dealing with multimorbidity and their overview of patient’s life, it is due to stress that GPs play an important role in achieving a life time perspective on multimorbidity patterns [52]. Hence, primary care providers are in urgent need of more knowledge on the trajectories of multimorbidity in patient’s life.

## Conclusion

There are many challenges facing multimorbidity research, including the implementation of a longitudinal, life-time perspective from a family practice perspective. The present study, although exploratory by nature, has indicated some interesting features of the dynamics multimorbidity, which might provide avenues for research. This requires a further analysis of time patterns and life periods in patients’ lives. Future research is required for better understanding the trajectories of severe forms of multimorbidity, and might help to enhance the quality of the general practitioner’s contribution to the care of patients with multimorbidity. GP’s need to understand that their practice contains several groups of patients with different multimorbidity patterns. A very small proportion of patients has a very high number of chronic health problems (MM11+) and keeps adding health problems in their life. Furthermore, GP’s need to realise that more than one third of their patients accumulate four or more chronic health problems (MM4-5 and MM6-10) in their lifetime.

## Abbreviations

GP: General practitioner
ICD: International classification of diseases
ICPC: International Classification of Primary Care
ICHPPC: International Classification of Health Problems in Primary Care
MM: Multimorbidity
RNH: RegistratieNet Huisartspraktijken (Registration Network Family Practices)
WHO: World Health Organization
WONCA: World Organization of Family Doctors
